# Cardiovascular RiskprofilE - IMaging and gender-specific disOrders (CREw-IMAGO): rationale and design of a multicenter cohort study

**DOI:** 10.1186/s12905-017-0415-x

**Published:** 2017-08-07

**Authors:** Gerbrand A. Zoet, Cindy Meun, Laura Benschop, Eric Boersma, Ricardo P.J. Budde, Bart C.J.M. Fauser, Christianne J.M. de Groot, Aad van der Lugt, Angela H.E.M. Maas, Karl G.M. Moons, Jeanine E. Roeters van Lennep, Jolien W. Roos-Hesselink, Eric A.P. Steegers, Bas B. van Rijn, Joop S.E. Laven, Arie Franx, Birgitta K. Velthuis

**Affiliations:** 1 Wilhelmina Children’s Hospital Birth Center, University Medical Center Utrecht, Lundlaan 6, 3508 AB Utrecht, The Netherlands; 2 Division of Reproductive Medicine, Department of Obstetrics and Gynaecology, Erasmus Medical Center, ‘s-Gravendijkwal 230, 3015CE, Rotterdam, The Netherlands; 3 Department of Obstetrics & Gynaecology, University Medical Center Rotterdam, Erasmus MC, ‘s-Gravendijkwal 230, 3015CE, Rotterdam, The Netherlands; 4 Department of Cardiology, Erasmus Medical Center, ‘s-Gravendijkwal 230, 3015CE, Rotterdam, The Netherlands; 5 Department of Radiology, Erasmus Medical Center, ‘s-Gravendijkwal 230, 3015CE, Rotterdam, The Netherlands; 6 Department of Reproductive Medicine & Gynaecology, University Medical Center Utrecht, Heidelberglaan 100, 3584 CX Utrecht, The Netherlands; 7 Department of Obstetrics and Gynecology, VU University Medical Center, De Boelelaan 1117, 1081 HV Amsterdam, The Netherlands; 8 Department of Cardiology, Radboud University Medical Center, Geert Grooteplein-Zuid 10, 6525 GA Nijmegen, The Netherlands; 9 Julius Center for Health Sciences and Primary Care, University Medical Center Utrecht, Heidelberglaan 100, 3584 CX Utrecht, The Netherlands; 10 Department of Internal Medicine, Erasmus Medical Center, ‘s-Gravendijkwal 230, 3015CE, Rotterdam, The Netherlands; 11 Academic Unit of Human Development and Health, University of Southampton, Princess Anne Hospital, Coxford Road, Southampton, SO16 5YA UK; 12 Department of Radiology, University Medical Center Utrecht, Heidelberglaan 100, 3584 CX Utrecht, The Netherlands

**Keywords:** Reproductive disorders, Hypertensive pregnancy disorders, Polycystic ovarian syndrome, Primary ovarian insufficiency, Cardiovascular risk factors, Cardiovascular disease, CT angiography, Coronary artery calcium, Carotid siphon calcification, Cell-based biomarkers

## Abstract

**Background:**

Reproductive disorders, such as polycystic ovary syndrome (PCOS), primary ovarian insufficiency (POI) and hypertensive pregnancy disorders (HPD) like pre-eclampsia (PE), are associated with an increased risk of cardiovascular disease (CVD). Detection of early signs of cardiovascular disease (CVD), as well as identification of risk factors among women of reproductive age which improve cardiovascular risk prediction, is a challenge and current models might underestimate long-term health risks. The aim of this study is to assess cardiovascular disease in patients with a history of a reproductive disorder by low-dose computed tomography (CT).

**Methods:**

Women of 45 - 55 years, who experienced a reproductive disorder (PCOS, POI, HPD), are invited to participate in this multicenter, prospective, cohort study. Women will be recruited after regular cardiovascular screening, including assessment of classical cardiovascular risk factors. CT of the coronary arteries (both coronary artery calcium scoring (CACS), and contrast-enhanced coronary CT angiography (CCTA)) and carotid siphon calcium scoring (CSC) is planned in 300 women with HPD and 300 women with PCOS or POI. In addition, arterial stiffness (non-invasive pulse wave velocity (PWV)) measurement and cell-based biomarkers (inflammatory circulating cells) will be obtained.

**Discussion:**

Initial inclusion is focused on women of 45 - 55 years. However, the age range (40 - 45 years and/or ≥ 55 years) and group composition may be adjusted based on the findings of the interim analysis. Participants can potentially benefit from information obtained in this study concerning their current cardiovascular health and expected future risk of cardiovascular events. The results of this study will provide insights in the development of CVD in women with a history of reproductive disorders. Ultimately, this study may lead to improved cardiovascular prediction models and will provide an opportunity for timely adjustment of preventive strategies. Limitations of this study include the possibility of overdiagnosis and the average radiation dose of 3.5 mSv during coronary and carotid siphon CT, although the increased lifetime malignancy risk is negligible.

**Trial registration:**

Netherlands Trial Register, NTR5531. Date registered: October 21st, 2015.

## Background

Reproductive disorders, including polycystic ovary syndrome (PCOS), primary ovarian insufficiency (POI) and hypertensive pregnancy disorders (HPD) such as pre-eclampsia (PE), are associated with an increased risk of cardiovascular diseases (CVD).

### Polycystic ovary syndrome

Polycystic ovary syndrome (PCOS) has a prevalence of around 8 to 10% in Caucasian women and is the most common endocrine disorder in women of reproductive age [1]. According to the Rotterdam consensus criteria, PCOS is diagnosed when at least two of the following criteria are present: (i) oligo−/anovulation, (ii) clinical and/or biochemical hyperandrogenism, and (iii) polycystic ovaries on ultrasonography [2]. Insulin resistance, dyslipidemia and type 2 diabetes mellitus (T2DM) have been associated with PCOS [3–7].

Increasingly PCOS has been associated with cardiovascular risk factors, such as impaired glucose tolerance, obesity, metabolic syndrome (MetS) and hypertension. Several studies have ascertained premature signs of subclinical arterial disease in women with PCOS, such as abnormal carotid intima media thickness on ultrasound or coronary artery calcification score (CACS) on computed tomography (CT) [8–10]. Nevertheless, evidence on the potential association between PCOS and CVD endpoints is still limited [11–13].

### Primary ovarian insufficiency

Primary ovarian insufficiency (POI), formerly known as premature ovarian failure, is characterized by secondary amenorrhea for at least 4 months accompanied by elevated FSH levels above 40 IU/L, before 40 years of age [14]. The incidence of POI is reported to be 1-2% [15, 16]. POI is associated with elevated gonadotropins, hypoestrogenemia and hypoandrogenemia.

Early age at menopause, including POI, is associated with an increased incidence of coronary heart disease and CVD mortality [17–19]. Epidemiological data showed that the relative risk (RR) on CVD was 1.03 (95% confidence interval (CI) 1.01 – 1.05) for each 1- year decrease in age at menopause [19]. Hypoandrogenemia in women has been associated with an increased risk of atherosclerosis, as measured by CIMT or catheter angiography [20–23] and CVD events [24]. A recent systematic review and meta-analyses identified POI as an independent, modest risk factor for developing or dying from IHD (ischemic heart disease) (hazard ratio (HR) 1.69, 95% CI 1.29-2.21, *p* = 0.0001) and total CVD (HR 1.61, 95% CI 1.22-2.12, *p* = 0.0007) [25]. No relationship was found for POI and stroke (HR 1.03, 0.88 - 1.19, *p* = 0.74). These findings may implicate a decreased cardiovascular health in women with POI. However, like PCOS, it remains unclear to which extent POI is independently associated with CVD due to the paucity of data.

### Hypertensive pregnancy disorders

HPD include pregnancy-induced hypertension (PIH), PE and the hemolysis, elevated liver enzymes, low platelets (HELLP) syndrome. Together, this group of disorders complicates 5-12% of all pregnancies worldwide, [26] while PE alone is seen in 3-5% of all pregnancies [27, 28]. Several studies showed that both classical CVD risk factors and novel serum biomarkers for CVD were increased in former hypertensive pregnancies (PIH, late-onset PE and especially early-onset PE) compared to normotensive pregnancies in both premenopausal and postmenopausal women [29, 30]. Major CVD risk factors (e.g. hypercholesterolemia, hypertension, diabetes and MetS) were 3-4 fold more prevalent in formerly pre-eclamptic patients when compared with healthy controls of the same age at one to three years after index pregnancy [31–33]. However, mainly due to the relative young age (mean 30.5 years), the 10-year absolute risk of a CVD event as estimated by the Framingham Risk Score (FRS) was still low (mean estimated 10-year cardiovascular disease risk 1.08%) [31].

Women who were diagnosed with pre-eclampsia have a twofold future CVD risk [34–38]. The relative risk of developing hypertension later in life in women with a history of pre-eclampsia is 3.74 [39]. Moreover, the risk of developing diabetes later in life is also 2-3 times increased in women with a history of pre-eclampsia compared to women without such a history [40–42]. These findings have led to the hypothesis that pregnancy acts as a stress-test for CVD later in life [43].

The sub-analyses of a longitudinal follow-up study of the HYPITAT trial – the HyRAS study – showed neither significant differences in hypertension and biochemical cardiovascular risk factors postpartum, nor a difference in the estimated 10- and 30 year Framingham cardiovascular event risk, between women with a history of late-onset pre-eclampsia compared to women with PIH [32]. On the other hand, the increased CVD risk does appear to be more pronounced in the subgroup of early-onset of pre-eclampsia (generally defined as pre-eclampsia occurring before 34 weeks of gestation), with a RR of 7 to 8 on IHD and death due to IHD [34, 35].

Despite recent advances in long-term follow-up after reproductive disorders, identifying women at increased risk for premature CVD remains a challenge. The use of the FRS and other risk models for IHD, like the Systematic Coronary Risk Evaluation (SCORE), Reynolds risk score and the Pooled Cohort Equations, are limited by their underestimation of lifetime CVD risk in young women. The recently published Dutch guideline “Cardiovascular Risk Management after Reproductive Disorders” recommends all women with a history of a reproductive disorder to optimize lifestyle factors [44]. Patients with a history of pre-eclampsia are advised to generate a risk profile at 50 years of age, as their risk of hypertension and diabetes mellitus is increased and the onset of these cardiovascular risk factors is up to 7 years earlier compared to women without pregnancy complications [39, 45]. However, longitudinal follow-up data on biomarkers, signs and symptoms of premature subclinical atherosclerosis are needed to better identify the potential adverse effects of female-specific risk factors and life-events on CVD risk.

### Serum biomarkers

Circulating endothelial cells, extracellular vesicles and circulatory inflammatory cells might lead to discovery of new biomarkers for women with reproductive disorders which are at risk for CVD development.

Both HPD and CVD later in life share a pathophysiologic pathway of vascular (endothelium) damage. Pre-eclampsia is associated with an increased number of circulating endothelial cells due to a high degree of endothelial cell activation or injury. Extracellular vesicles (e.g. microvesicles, exosomes) reflect the disease state of pre-eclampsia patients compared to healthy pregnant women [46, 47]. In addition, extracellular vesicles -associated polygenic immunoglobin receptor, cystatin C, and complement factor C5a are markedly increased in patients suspected of acute coronary syndrome [48]. As EVs might be involved in both reproductive disorders and CVD, they could possibly serve as a biomarker.

The inflammatory profile of circulating cells is proven to be very different in women suffering from CVD [47]. For example, carotid plaques show sex-dependent inflammatory cell content, including neutrophils [49].

### Imaging

CACS acquired with CCT has been shown to have superior predictive value for CVD events to traditional risk factors, risk factor scores and serum biomarkers in asymptomatic persons [50]. Contrast-enhanced CCTA may have additional value over CACS as it can also identify non-calcified plaques, and thereby the total atherosclerotic burden, and assess the presence of coronary luminal narrowing [51–54]. As calcification of plaque occurs at a relatively late stage in atherosclerosis, significant coronary atherosclerosis may be visualized earlier by visualizing the non-calcified coronary plaque with CCTA. Data of CCT as a diagnostic tool, i.e. CACS or CCTA, is scarce and inconclusive for women with reproductive disorders.

Several studies have assessed the presence of coronary calcium in PCOS. Although some of these studies showed an increased CACS in women with PCOS, a recent large, cross-sectional study could not find an association with PCOS and CACS [55–58].

In a retrospective cohort study published in 2007, the relation between HPD and coronary calcification later in life was assessed in 491 women (mean age 66.8 years, standard deviation 5.4 years) [59]. Coronary calcifications (Agatston score ≥ 1) were found in 305 women (62.9%).This study showed that a self-reported history of hypertension during pregnancy is related to higher CACS in the 7th decade of life (Odds ratio (OR) 1.57, 95% CI 1.04 - 2.37). In an adjusted model correcting for age, BMI, waist:hip ratio, systolic blood pressure and diastolic blood pressure the relation did not reach statistical significance anymore (OR 1.52, 95% CI 0.96 - 2.39) [59]. Other retrospective studies assessing coronary artery disease (CAD) by catheter coronary angiography in women suspected for CAD and with a history of HPD showed contrasting results [60, 61]. A recent prospective cohort study conducted among 40 former pre-eclamptic women and 40 age- and parity-matched healthy controls showed increased CACS in former pre-eclamptic women at a mean age of 59.5 ± 4.6 years. The unadjusted OR for having higher CACS due to preeclampsia was 3.54 (95% CI 1.39 - 9.02), although the adjusted model correcting for BMI and hypertension did not reach statistical significance anymore [62].

There is mounting evidence that intracranial carotid artery calcifications are associated with increased large artery and intracranial artery stiffness, as well as ischemic stroke, white matter abnormalities and cognitive impairment [63, 64]. Recent studies found a relationship between carotid siphon calcium (CSC) and white matter hyperintensities and lacunar infarcts [65–67]. Other studies, however, could not confirm these findings [68–70].

Collectively, previous studies indicate an association between women with reproductive disorders and CVD later in life. Current risk profiles are inadequate to establish future CVD risk in still relative young premenopausal women. The aim of this study is to assess the diagnostic value of cell-based biomarkers, CCT imaging (both non-contrast CACS and contrast enhanced CCTA) and non-contrast CSC in patients with a reproductive disorder to detect CVD.

## Methods

### Study design and study setting

In this multicenter, prospective, cross-sectional study of patients with a reproductive disorder (PCOS, POI or HPD) we aim to assess the diagnostic value of cell-based biomarkers and CT imaging of the coronary arteries and carotid siphon in the detection of CVD. Patients will be invited to participate at their regular cardiovascular screening, which is performed at two large University Medical Centers in Utrecht (UMC Utrecht) and Rotterdam (Erasmus MC) in the Netherlands.

### Participant characteristics

All patients with a reproductive disorder undergo regular cardiovascular screening at a specialized vascular outpatient clinic in one of the participating hospitals as part of standard care for cardiovascular diseases. The study population consists of women aged 45-55 years within three different groups:

1. Women with PCOS defined by Rotterdam consensus criteria, requiring the presence of at least two of the following criteria: (i) oligo−/anovulation, (ii) clinical and/or biochemical hyperandrogenism, and (iii) polycystic ovaries on ultrasonography.
2. Women with POI defined as women with secondary amenorrhea for at least 4 months accompanied by elevated FSH levels above 40 IU/L, occurring prior to 40 years of age.
3. Women with a history of HPD (PIH, early-onset PE (i.e. delivery before 34 weeks of gestation) and late-onset PE (i.e. delivery after 34 weeks of gestation)) according to the ISSHP criteria, verified in clinical records.

Patients with any serious illness that can compromise study participation, patients with high risk for contrast nephropathy (renal dysfunction with an estimated glomerular filtration rate < 60 ml/min/1.73 m2) or patients with a history of myocardial infarction are excluded from the study.

After written informed consent is obtained, patients will undergo cardiovascular imaging assessment by CCT imaging, biomarkers and a non-invasive vascular measurement.

### Coronary CT imaging

CCT is performed using a multislice CT scanner (256 slice Philips CT, Philips Healthcare, Best, the Netherlands or dual source Somaton Force or Drive Siemens CT, Siemens, Forchheim, Germany) with prospective ECG-triggering. A non-contrast coronary CT is acquired first to calculate the CACS (scan parameters 120 kV, 50 mAs or reference mAs of 80 mAs). Participants with a heart rate > 65 beats/min may receive an oral (25-50 mg) and/or intravenous (5-20 mg) beta-blocker (metoprolol, Selokeen AstraZeneca, Zoetermeer, the Netherlands) before the scan. All participants will receive sublingual nitroglycerine just before the CCTA. CCTA scan parameters will be as follows depending on the participant’s weight:

1. For the Philips scanner a prospective ECG-triggered acquisition is performed at a mid-diastolic phase (78%) with 80-120 kV; 195-210 mAs; and 90–115 ml non-ionic contrast material (Iopromide, 300 mg I/ml; Ultravist, Bayer Healthcare, Berlin, Germany) followed by 30–40 ml saline, both injected at a speed of 6–6.7 ml/s. A bolus-tracking technique is used to time the arrival of contrast in the coronary arteries. The CCTA scan is initiated once a threshold of 130 HU is reached in the descending aorta followed by a 7-s post-threshold delay. CCTA’s are reconstructed with 0.9 mm slice thickness and iDose iterative reconstruction level 4 and 6.
2. For the Siemens scanners a sequential prospective ECG–triggered acquisition is performed with a pulsing window width depending on heart rate or a high-pitch acquisition timed to image the heart in diastole in case of low regular heart rate. KV and mAs are selected using automatic KV selection based on the topogram (range 70-120 kV) and a reference mAs setting of 230 mAs at 120 kV. At lower kVs reference mAs is automatically adapted accordingly. Either a bolus-tracking technique or test bolus injection with 10 ml contrast is used to time the arrival of contrast in the coronary arteries at the discretion of the technician. Non-ionic contrast material (Iopromide, 370 mg I/ml; Ultravist, Bayer Healthcare, Berlin, Germany) is used followed by 30-40 ml saline, both injected at a speed of 5.4 ml/s. Total contrast volume is calculated as scan time + 8 s multiplied by contrast flow rate. Mostly around 70 ml of contrast is injected for the CCTA. CCTA’s are reconstructed with 0.6 mm slice thickness and ADMIRE iterative reconstruction level 3.

The total CCT radiation dose to which participants will be exposed is expected to be within 3.0 mSv.

CT scans are post processed on a workstation (IntelliSpace Portal, Philips Healthcare, QAngio CT software, Medis Medical Imaging or SyngoVia,Siemens) by experienced personnel. CACS is measured on the non-contrast CT with the Agatston scoring method [71]. Coronary artery calcium is defined as a density of >130 Hounsfield units (HU) in a coronary artery. Total CACS is calculated by the sum of all lesions in all four coronary arteries and their side branches. The total Agatston score will be categorized as no calcification (CACS = 0), mild (CACS > 0 and < 100), moderate (CACS ≥ 100 and < 400) and severe (CACS ≥ 400) calcification; and compared with the MESA database [72]. Semi-automated vessel analysis is used to make multiple curved multiplanar reconstructions (MPR) of all coronary arteries on the CCTA data.

All cardiac CT scans will be assessed by an experienced cardiovascular radiologist in both academic hospitals. Image quality, plaque characteristics and coronary lumen stenosis will be analyzed on a 18-segment basis according to the modified American Heart Association classification [73, 74]. Plaque composition will be evaluated in a qualitative manner as calcified, mixed (both calcified and non-calcified components) and non-calcified (plaques without calcium). Total atherosclerotic plaque burden will be measured with both the segmental involvement score (SIS) and the segment stenosis score (SSS) based on the 18-segment coronary artery model [74]. Luminal stenosis will be graded as absent, minimal (1 – 24%), mild (25 – 49%), moderate (50 – 69%), and severe (≥ 70%) narrowing on the basis of diameter measurements comparing the diameters of the maximal stenosis to a reference diameter proximal and distal to the stenotic area [75]. If severe calcifications are present and quantification of stenosis is difficult, the radiologist will refrain from stenosis quantification and score the segments involved as ‘calcified, stenosis unclear’.

### Carotid siphon calcification imaging

A non-contrast CT with 20-40 mm coverage is planned around the sella turcica to include the intracranial carotid siphon and anterior clinoid process. The head is tilted with the chin towards the breast to avoid scanning the eye lens. The CSC radiation dose to which participants will be exposed is expected to be less than 0.5 mSv on average. Scan parameters will be as follows:

1. For the Philips scanner: 20 mm coverage from petrous apex to ICA top with 120 kV, 100 mAs, brain filter B and C, iDose iterative reconstruction level 3, 1 mm and 2 mm reconstruction thickness without overlap.
2. For the Siemens scanner (Somatom Force or Drive): 40 mm coverage from horizontal part of the petrous ICA to ICA top with 120 kV, 100 mAs reference, H31s and H45s head filter, 075 mm reconstruction thickness and 0.4 mm increment.

The axial scans are visually assessed by a radiologist for presence or absence of carotid siphon calcification. The severity of CSC will be visually assessed and categorized according to Woodcock as 0 = absent, 1 = mild (thin, discontinuous), 2 = moderate (thin, continuous or thick, discontinuous) or 3 = severe (thick, continuous) [76, 77]. In addition, the CSC will be semi-automatically quantified as described in detail previously [65].

### Single center side study: Serum biomarkers and non-invasive vascular measurements

Classical cardiovascular biochemical risk factor assessment (glucose, insulin, triglycerides, total cholesterol, low-density lipoprotein cholesterol and c-reactive protein) and a general hematological profile (total red and white blood cells and differential counts of nucleated cells) will both be determined.

As part of a single center side study in the UMC Utrecht, the neutrophil, monocyte, and lymphocyte cell numbers and subtype distribution will be examined based on established cell surface marker expression by flow cytometry analysis [78, 79]. Peripheral blood mononuclear cells and circulating endothelial cells will be isolated and sorted directly after drawing blood. To assess histone modification in inflammatory genes, monocytes are cultured in the presence of atherosclerosis associated antigens (oxLDL, HSP). Plasma will be used to isolate and detect (endothelial) microparticles. Proteomics on isolated microparticles will be performed on selected samples and analyzed. Lupus anticoagulants are phospholipid-dependent coagulation inhibitors that are detected with a functional assay based on a clotting test. Platelet reactivity can be assessed with the platelet activation test (PACT), a whole blood functional test of the total platelet response capacity.

Arterial stiffness measured by pulse wave velocity (PWV) is a predictor of cardiovascular events. The Arteriograph (TensioMed, Budapest, Hungar. EC Directive 93/42/EEC, ISO 13485:2003, ISO 13485:2004) is an oscillometric arterial stiffness measurement, which will be performed directly before the CCT in the UMC Utrecht.

### Outcome

The primary outcome is the presence of relevant CAD that will be defined as one or more of the following on CT: a CACS ≥100 AU or luminal stenosis ≥ 50%. All measurements will be discussed with participants individually under supervision of a vascular specialist. For management of cardiovascular risk factors, the current guideline on European Guidelines on cardiovascular disease prevention will be used [80]. All patients with relevant CAD are discussed with a cardiologist. Management and treatment decisions are left to the discretion of the cardiologist. As a general rule, participants with a CACS of 100-400 AU without obstructive CAD will receive lifestyle advice and may be recommended to initiate treatment with statins [81, 82]. Participants with a CACS ≥ 400 AU or coronary artery stenosis ≥ 50% will be offered a consultation with a cardiologist to discuss management options. Other relevant cardiac findings will be discussed with the cardiologist. If incidental extra-cardiac findings, such as lung or liver lesions, are considered to be of clinical importance, recommendations of further testing, follow-up or referral to another specialist will be made.

### Sample size calculation

No prospective studies have performed CCT (including both CACS and CCTA) in women with reproductive disorders as part of long-term cardiovascular follow up. The estimation of the expected necessary sample size is based on α = 0.05 and desired power = 0.90. In addition, the background prevalence of coronary plaque (both non-calcified and calcified) in asymptomatic, healthy females ≥ 45 years old based on the CCTA study by Kim et al. (2013) is estimated 6.7% [83]. We presume a relative risk of around 2 for the development of CVD is women with reproductive disorders [44]. This leads to an (conservative) estimated prevalence of coronary artery disease (plaque) of 13.4% (95% CI 10.0 - 17.6) and a minimal sample size of at least 261 CCT’s in both the HPD and the PCOS/POI group.

Based on these results and given the radiation-induced health risks, initial inclusion will be confined to women who are 45-55 years old and we will perform an interim analysis after 300 CCT’s (100 in patients with HPD, 100 in patients with PCOS and 100 in patients with POI). If the prevalence of plaque as seen on CCTA is ≥ 10%, which is the lower bound of the 95% CI in our estimated prevalence of coronary artery disease, we expect to find significant differences compared to controls. In that case, we will perform the remaining 300 CCT’s (200 in patients with HPD and 100 in patients with PCOS/POI), leading to a total of 300 CCT’s in patients with HPD and 300 CCT’s in patients with PCOS/POI. The age range (40-45 years and/or ≥ 55 years) and group composition for later inclusion may be adapted based on the findings of the interim analysis. If the prevalence of plaque as seen on CCT is ≤ 10%, we do not expect to find any significant differences compared to controls and therefore we will withdraw the remaining CCTA’s and focus on CACS only.

### Statistical analysis

Data analysis will be performed using SPSS. A probability (p) less than 0.05 will be considered significant. The characteristics of participants will be described using means +/− standard deviations for continuous variables using a two tailed independent t-test for comparison between groups (e.g. plaque burden). Categorical variables will be expressed as numbers (percentages) or medians (with inter quartile ranges) and compared with a chi-square test (e.g. CACS percentiles). Identical tests will be performed for circulating biomarkers. If several variables are identified to be statistically associated with cardiovascular risk factors, multiple linear regression or multiple logistic regression will be performed to identify the most important associations, appropriate to the endpoint chosen.

### Ethical considerations

This study has been approved by the Medical Research Ethics Committee of the University Medical Center Utrecht (MEC number 15-508). This trial is registered in the Dutch Trial Register, NTR 5531, http://www.trialregister.nl/trialreg/admin/rctview.asp?TC=5531, date of registration: October 21st, 2015.

## Discussion

In this multicenter, prospective, cross-sectional study in patients with a reproductive disorder (PCOS, POI or HPD) we aim to assess CVD by CCT imaging (both CACS and CCTA) and CSC. Given the increased risk on CVD later in life, we believe the low radiation exposure is justified. Moreover, a substudy of the association of cell-based biomarkers and CCT will be performed.

The results of this study will provide insights in the added value CT imaging in the detection of cardiovascular disease. Ultimately, these insights can lead to improved cardiovascular prediction models in these women and may provide an opportunity for adjusted preventive strategies.

## Abbreviations

CACS: Coronary artery calcium scoring
CCT: Coronary computed tomography
CCTA: Contrast-enhanced CT coronary angiography
CI: Confidence interval
CSC: Carotid siphon calcium
CVD: Cardiovascular diseases
FRS: Framingham Risk Score
HELLP: Hemolysis, elevated liver enzymes, low platelets syndrome
HPD: Hypertensive pregnancy disorders
HR: Hazard ratio
MetS: Metabolic syndrome
OR: Odds ratio
PCOS: Polycystic ovary syndrome
PIH: Pregnancy induced hypertension
POI: Primary ovarian insufficiency
PWV: Pulse wave velocity
RR: Relative risk
SCORE: Systematic Coronary Risk Evaluation
